# Anxiety and depression prevalence and associated factors in patients with knee osteoarthritis

**DOI:** 10.3389/fpsyt.2024.1483570

**Published:** 2025-01-24

**Authors:** Qizhen Feng, Mingjie Weng, Xi Yang, Min Zhang

**Affiliations:** ^1^ Department of Osteoarthritis, The Second Hospital of Shanxi Medical University, Taiyuan, China; ^2^ Department of Nutrition and Food Hygiene, School of Public Health, Shanxi Medical University, Taiyuan, China; ^3^ The First Clinical College of Shanxi University of Chinese Medicine, Jinzhong, China

**Keywords:** knee osteoarthritis, anxiety, depression, social support, quality of life

## Abstract

**Objective:**

With the evolving spectrum of diseases, psychological conditions such as anxiety and depression have emerged as significant global public health concerns. Notably, these psychological disorders are prevalent among patients suffering from knee osteoarthritis (KOA). Consequently, this study included 360 hospitalized patients diagnosed with KOA to examine their levels of anxiety and depression and to analyze the factors influencing these psychological states.

**Methods:**

A cohort of KOA patients from the Second Hospital of Shanxi Medical University was assessed using a general condition questionnaire, European five-dimensional health status scale (EQ5D), Western Ontario and McMaster University Osteoarthritis (WOMAC), Social Support Rating Scale (SSRS), and Hospital Anxiety and Depression Scale (HADS). Logistic regression analysis was employed to identify the factors affecting anxiety and depression.

**Results:**

Among 360 patients with KOA, 28.06% experienced anxiety, and 30.27% experienced depression. Multivariate logistic regression analysis showed that lower BMI, QOL, and utilization of social support scores are risk factors for anxiety and depression in KOA patients (*P*<0.05). Additionally, in patients with KOA, younger age, lower subjective support, and higher scores in function and daily activities emerged as significant risk factors for depression (*P*<0.05).

**Conclusion:**

Anxiety and depression in patients with KOA warrant significant attention due to their impact on overall well-being. The factors influencing these mental health conditions are multidimensional. In clinical practice, it is essential to integrate these various influencing factors to develop targeted mental health care services. By doing so, healthcare providers can enhance the overall mental health and QOL for individuals suffering from KOA.

## 1 Introduction

Knee osteoarthritis (KOA) is a prevalent chronic condition, particularly among individuals over 50 years of age (1). The primary pathological features of this disease include the wear of articular cartilage, narrowing of the joint space, and remodeling of the subchondral bone (2). Patients typically experience clinical symptoms such as pain, stiffness, and movement disorders (3). Despite the numerous treatments available for KOA (4), effective options to delay disease progression remain lacking. Chronic pain and daily living difficulties significantly diminish patients’ quality of life (QOL) and increase their psychological burden, leading to varying degrees of anxiety, depression, and other psychological issues (5–7).

Research has shown a significant correlation between KOA and psychological issues such as anxiety and depression. Individuals with KOA have a higher prevalence of anxiety and are two to three times more likely to exhibit depressive symptoms compared to the general population (8, 9). Research indicates that the prevalence of anxiety and depression among patients with KOA varies significantly across different countries. In the United States, the prevalence is reported to be 11.1% (10), whereas in the Netherlands, it is higher at 25.7% (11). Notably, in China, the prevalence rates of anxiety and depression were 29.2% and 37.5% (12), respectively exceeding those in the USA and the Netherlands. Additionally, studies have identified specific demographic factors that increase the likelihood of developing depression among KOA patients (13). These factors include being female, younger in age, having a higher body mass index (BMI), and possessing a lower level of education (14). Emotional problems such as depression and anxiety are recognized as risk factors that may accelerate the progression of KOA. Previous research indicates that anxiety and depression can lead to physical dysfunction, which in turn affects patients’ mobility. This reduction in physical activity can hinder pain and inflammation relief, potentially trapping KOA patients in a cyclical pattern of pain (15, 16). Moreover, studies have found a positive correlation between the severity of depression and the intensity of pain symptoms in KOA patients. Depression symptom not only has the potential to accelerate the progression of KOA but may also exacerbate other psychological issues (17).

At present, the relationship between anxiety, depression, and KOA has garnered significant attention in the medical community (18). Therefore, The purpose of this study was to explore the current situation of anxiety and depression in the KOA population and analyze its influencing factors so as to provide a theoretical basis for the subsequent formulation of targeted psychotherapy programs to reduce the occurrence of anxiety and depression in the KOA population.

## 2 Methods

### 2.1 Participants

This study is a cross-sectional study. This study involved patients with KOA who visited the Osteoarthritis Department at the Second Hospital of Shanxi Medical University from June to August 2024. A total of 375 patients met the inclusion and exclusion criteria. The diagnostic criteria for primary KOA included: (1) The patient had experienced recurrent knee pain in the past month. (2) An X-ray taken in a standing or weight-bearing position should reveal narrowing of the joint space, sclerosis of the subchondral bone, cystic degeneration, or osteophyte formation at the joint edge. (3) Synovial fluid analysis, conducted at least twice, should show transparent and viscous fluid with a white blood cell count of less than 200/ml. (4) Elderly patients aged 40 years and older. (5) The presence of morning stiffness lasting no more than three minutes. (6) Bone fricative or bone rubbing sensation when moving. A diagnosis of KOA can be confirmed if the criteria of either (1) and (2), or (1), (3), (5), and (6), or (1), (4), (5), and (6) are satisfied.

**Figure 1:**
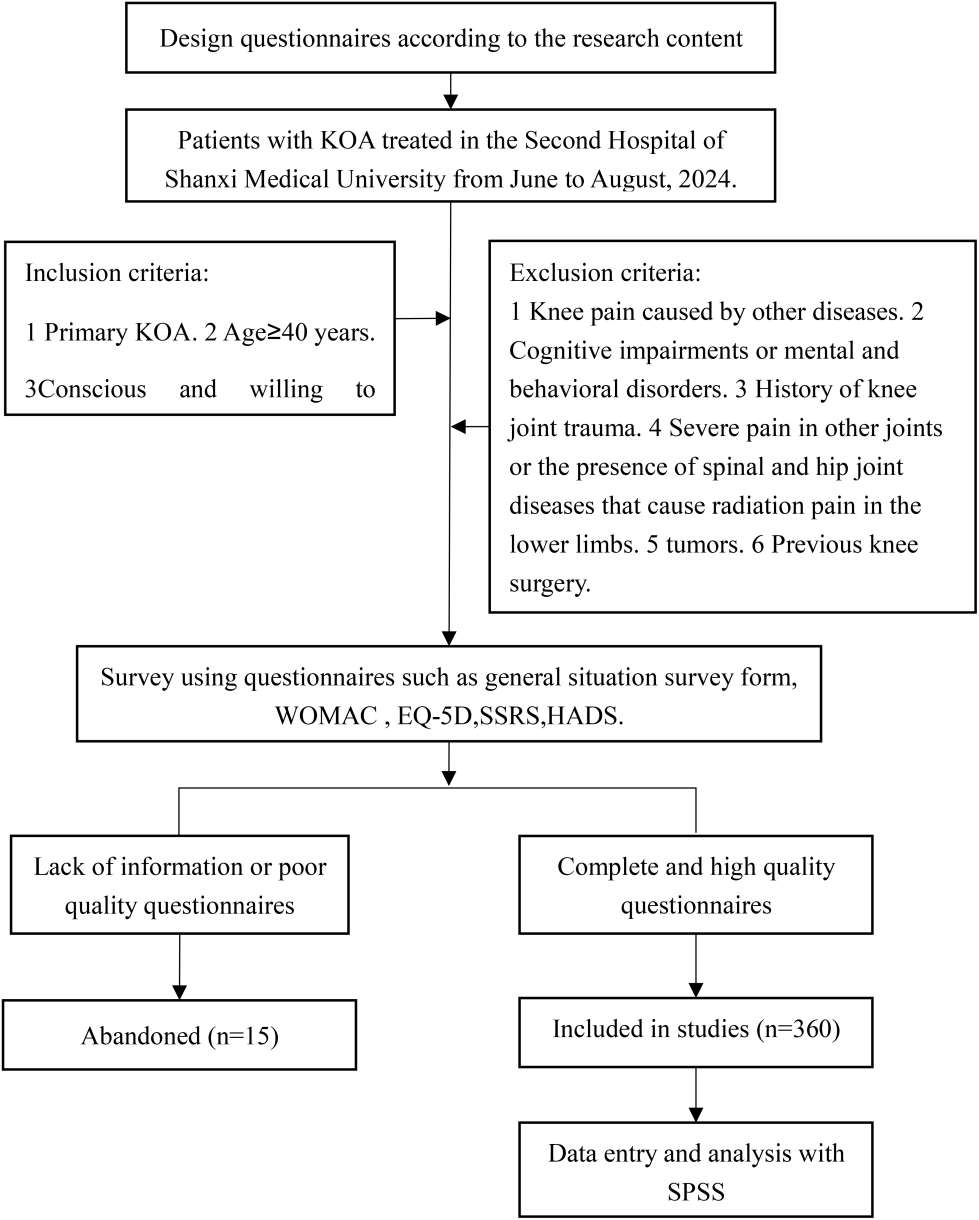
Study flow chart.

Inclusion criteria: (1) Complies with diagnostic criteria for primary KOA. (2) Age 40 years or older. (3) The ability to provide conscious and voluntary consent to participate in this study. Exclusion criteria: (1) Knee joint pain attributed to conditions other than osteoarthritis, such as rheumatoid arthritis or osteonecrosis. (2) Cognitive impairments or mental and behavioral disorders. (3) History of knee joint trauma, including sprains, contusions, or other injuries. (4) Severe pain in other joints or the presence of spinal and hip joint diseases that cause radiation pain in the lower limbs. (5) Systemic malignant tumors or tumors located around the knee joints. (6) Previous knee surgery. A flowchart detailing patient inclusion and exclusion is shown in **Figure 1**.

### 2.2 Data collection

This study employed a cross-sectional design, wherein basic demographic and clinical information were gathered through a structured questionnaire. For patients who met the inclusion and exclusion criteria, the investigator provided a detailed explanation of the study’s purpose and content to both the patients and their family members. Upon obtaining informed consent, the investigator administered the questionnaire on the first day of the patient’s admission, prior to the commencement of treatment.

### 2.3 Measurement tools

(1)General situation survey form: The general situation questionnaire was self-designed, including gender, age, BMI, education, marital status, place of residence, monthly per capita family income, medical payment method, occupation, the number of affected knee joints, disease duration, and the presence of swelling in the affected knee joint. (2) The WOMAC is a valuable tool for assessing the severity and treatment effects of KOA. This scale encompasses three dimensions: pain, stiffness, and functional ability in daily activities. It comprises 24 items addressing the fundamental symptoms and signs of KOA and can be completed quickly. Higher scores reflect poorer knee function (19). (3) The EQ-5D is a tool used to assess the health status of a population. It comprises two main components: a questionnaire and a utility conversion table. The questionnaire evaluates five dimensions of health: mobility, self-care ability, daily activity ability, pain or discomfort, and anxiety or depression. Each dimension allows respondents to report their status as “no problem,” “moderate problem,” or “severe problem.” After completing the questionnaire, the responses are converted into a score using the utility conversion table. A score closer to 1 indicates a higher QOL. (4) The SSRS is a valuable tool for assessing the social support experienced by individuals. This scale comprises ten items that evaluate three dimensions: objective support, subjective support, and the utilization of social support. Objective support encompasses tangible, visible, or actual support, such as direct material assistance, as well as the presence and involvement of social networks and group affiliations. Subjective support, on the other hand, pertains to the emotional experiences of individuals, precisely their feelings of being respected, supported, and understood within society. The utilization of the social support dimension examines the variations in how individuals make use of the social support available to them. The cumulative scores from these three dimensions are interpreted as follows: a total score of less than 20 indicates low social support, a score between 20 and 30 signifies general social support and a score greater than 30 reflects satisfactory social support.

(5) The HADS is a self-reported scale used to screen and assess anxiety and depressive moods in general hospital patients, especially those experiencing symptoms due to physical illness. It demonstrates good internal consistency and test-retest reliability (20). The assessment tool comprises 14 items, with seven items dedicated to evaluating depression and the other seven focused on assessing anxiety. The anxiety scale is denoted by ‘A,’ while the depression scale is represented by ‘D.’ Each item is rated on a 4-point scale ranging from 0 to 3. For each subscale, a total score between 0 and 7 indicates the absence of anxiety or depression, whereas a score of 8 or higher signifies the presence of anxiety or depression (21).

### 2.4 Sample size

The sample size typically ranges from five to ten times the number of independent variables. In this study, A total of 15 factors were used as a basis, including 12 factors such as age, gender, and education level from a general situation questionnaire, as well as three scoring scales including WOMAC, SSRS, and EQ-5D. To account for the influence of response rates, we expanded the sample size by 10%. Using the formula: sample size = number of independent variables × 10/(1 - 10%), we calculated the required sample size to be 167.

### 2.5 Statistical analysis

The data were analyzed using SPSS 27.0 statistical software. Measurement data were presented as mean ± standard deviation while counting data were expressed as frequency and percentage (%). Continuous variables were analyzed using either the t-test or the Mann-Whitney U test. Categorical variables underwent univariate analysis through the chi-square test or Fisher’s exact test. For multivariate analysis, logistic regression was employed. A p-value of less than 0.05 was considered statistically significant.

## 3 Results

### 3.1 General demographic and disease characteristics

A total of 375 questionnaires were distributed, resulting in the recovery of 360 valid responses, yielding an effective recovery rate of 96%. Among the valid questionnaires, 92 respondents were male, and 268 were female. The average age of participants was 64.02 years, with a mean BMI of 25.52 kg/m². The predominant marital status is married. Regarding education levels, 243 participants had completed junior high school or lower, 94 had attended high school or junior college, and 23 had obtained a university degree or higher. The majority are either retired or unemployed. The residential distribution included 181 urban residents and 179 rural residents. Families with a monthly per capita income below 2000 yuan constitute a significant proportion. Regarding medical payment methods, 350 respondents had medical insurance. See **Table 1** for specific demographic characteristics and disease distribution.

**Table 1:** General demographic and disease characteristics.

Project	Category	Cases(n)	Constituent ratio (%)
Gender	Male	92	25.56
	Female	268	74.44
Age, years	40-49	19	5.28
	50-59	82	22.78
	60-69	166	46.11
	≥70	93	25.83
BMI, kg/m2	18.4	7	1.94
	18.4-24.9	148	41.11
	≥25	205	56.95
Marital status	Unmarried	1	0.28
	Married	316	87.78
	Widowed	43	11.94
Education	Junior high school or lower	243	67.50
	High school or junior college	94	26.11
	University degree or higher	23	6.39
Residence	Urban	181	50.28
	Rural	179	49.72
Professional status	Employed	36	10
	Retired or unemployed	324	90
Income	<2000	190	52.78
	2000-5000	131	36.39
	>5000	39	10.83
Complication	Hypertension	166	46.11
	Diabetes mellitus	56	15.56
	Coronary atherosclerotic heart disease	13	3.61
Medical insurance	Yes	350	97.22
	No	10	2.78
Number of affected knees	Unilateral	244	67.78
	Bilateral	116	32.22
Duration, years	<5	175	48.61
	5-10	99	27.5
	>10	86	23.89
Joint swelling	Yes	115	31.94
	No	245	68.06

### 3.2 Anxiety and depression in patients with KOA

In this study involving 360 patients with KOA, anxiety was identified in 101 patients. Additionally, depression was observed in 109 cases.

### 3.3 The QOL and social support of patients with KOA

The QOL score for patients with KOA was 0.81 ± 0.08, with a maximum possible score of 1, representing the ideal state, and a minimum score of 0.48. Among the 360 KOA patients surveyed, none reported having low social support, while 64 patients had general social support, and 296 patients reported satisfactory social support. Patients with anxiety or depression scored significantly lower in all dimensions of the social support scale compared to those without these conditions (*P*<0.05). Furthermore, patients with satisfactory social support had lower scores on the WOMAC, as well as lower anxiety and depression scores, compared to those with general social support (*P*<0.05). For further details, refer to **Tables 2** and **3**.

**Table 2:** Social support for patients with KOA(Mean±SD).

	Anxiety			Depression		
	Yes	No	*P* value	Yes	No	*P* value
Objective support	7.72±1.49	7.97±2	0.20	7.42±1.59	8.11±1.95	<0.05
Subjective support	20.24±4.53	22.73±4.73	<0.05	20.28±4.59	22.79±4.7	<0.05
Utilization of social support	6.85±2.09	7.78±2	<0.05	6.7±2.03	7.88±1.98	<0.05
Total scores	34.5±6.28	38.45±6.79	<0.05	34.23±6.25	38.69±6.68	<0.05

**Table 3:** The WOMAC score, anxiety and depression of KOA patients with different social support levels(Mean±SD).

	General social support	Satisfactory social support	*P* value
WOMAC	35.95±16.46	31.84±16.43	<0.05
Anxiety	6.88±3.07	5.06±3.25	<0.05
Depression	7.13±3.16	5.3±3.45	<0.05

### 3.4 Univariate analysis of anxiety and depression in patients with KOA

The findings indicate statistically significant differences in anxiety levels based on several factors, including BMI, education, residence, professional status, medical payment methods, pain severity, stiffness, function and daily activities, QOL, and utilization of social support (*P*<0.05). In contrast, no significant differences were observed concerning age, disease duration, or monthly income (*P*>0.05). Similarly, significant differences in depression levels were found in relation to age, BMI, education, residence, monthly income, medical payment methods, stiffness, function and daily activities, QOL, subjective support, and utilization of social support (*P*<0.05). However, no statistical differences were noted in occupation, disease duration, or pain severity (*P*>0.05). For further details, refer to **Tables 4** and **5**.

**Table 4:** Univariate analysis of anxiety in patients with KOA.

	Anxiety	Non-Anxiety	*P* value
Gender (n, %)			0.12
Male	20(21.74)	72(78.26)	
Female	81(30.22)	187(69.78)	
Ages(years)	64.51±7.7	63.83±9.11	0.33
BMI (kg/m^2^)	24.98±3.89	25.73±3.26	<0.05
Marital status (n, %)			0.76
Married	90(28.48)	226(71.52)	
Unmarried	0(0)	1(100)	
Widowed	11(25.58)	32(74.42)	
Education (n, %)			<0.05
Junior high school or lower	82(33.74)	161(66.26)	
High school or junior college	17(18.09)	77(81.91)	
University degree or higher	2(8.7)	21(91.3)	
Residence (n, %)			<0.05
Urban	36(19.89)	145(80.11)	
Rural	65(36.31)	114(63.69)	
Professional status (n, %)			<0.05
Employed	2(5.56)	34(94.44)	
Retired or unemployed	99(30.56)	225(69.44)	
Income (n, %)			0.09
<2000	62(32.63)	128(67.37)	
2000-5000	32(24.43)	99(75.57)	
>5000	7(17.95)	32(82.05)	
Medical insurance (n, %)			0.89
Yes	98(28)	252(72)	
No	3(30)	7(70)	
Number of affected knees (n, %)			0.52
Unilateral	71(31.7)	173(68.3)	
Bilateral	30(25.86)	86(74.14)	
Duration (years)			0.72
<5	48(27.43)	127(72.57)	
5-10	2(26.26)	73(73.74)	
>10	27(31.4)	59(68.6)	
Joint swelling (n, %)			0.15
Yes	38(33.04)	77(66.96)	
No	63(25.71)	182(74.29)	
WOMAC			
Pain	9.2±3.81	6.78±3.5	<0.05
Stiff	2.88±1.73	2.17±1.75	<0.05
Function and daily activities	29.23±12.82	20.14±11.31	<0.05
EQ-5D	0.75±0.09	0.82±0.08	<0.05
SSRS			
Objective support	7.72±1.49	7.97±2	0.72
Subjective support	20.24±4.53	22.73±4.73	0.12
Utilization of social support	6.85±2.09	7.78±2	<0.05

**Table 5:** Univariate analysis of depression in patients with KOA.

	Depression	Non- Depression	*P* value
Gender (n, %)			0.31
Male	24(26.09)	68(73.91)	
Female	85(31.72)	183(68.28)	
Ages(years)	64.3±7.65	63.9±9.17	<0.05
BMI (kg/m^2^)	24.83±3.64	25.81±3.34	<0.05
Marital status (n, %)			0.47
Married	93(29.43)	223(70.57)	
Unmarried	0(0)	1(100)	
Widowed	16(37.21)	27(62.79)	
Education (n, %)			<0.05
Junior high school or lower	88(63.79)	155(36.21)	
High school or junior college	19(20.21)	75(79.79)	
University degree or higher	2(8.7)	21(91.3)	
Residence (n, %)			<0.05
Urban	45(24.86)	136(75.14)	
Rural	64(35.75)	115(64.25)	
Professional status (n, %)			0.61
Employed	6(16.67)	30(83.33)	
Retired or unemployed	103(31.79)	221(68.21)	
Income (n, %)			<0.05
<2000	69(36.32)	121(63.68)	
2000-5000	33(25.19)	98(74.81)	
>5000	7(17.95)	32(82.05)	
Medical insurance (n, %)			0.47
Yes	107(30.57)	243(69.43)	
No	2(20)	8(80)	
Number of affected knees (n, %)			0.65
Unilateral	72(29.51)	172(70.49)	
Bilateral	37(31.9)	79(68.1)	
Duration (years)			0.36
<5	48(27.43)	127(72.57)	
5-10	30(30.3)	69(69.7)	
>10	31(36.05)	55(63.95)	
Joint swelling (n, %)			0.08
Yes	42(36.52)	73(63.48)	
No	178(72.65)	67(27.35)	
WOMAC			
Pain	8.57±3.7	6.98±3.66	0.1
Stiff	3.03±1.79	2.09±1.69	<0.05
Function and daily activities	28.78±12.44	20.05±11.47	<0.05
EQ-5D	0.76±0.09	0.82±0.07	<0.05
SSRS			
Objective support	7.42±1.59	8.11±1.95	0.15
Subjective support	20.28±4.59	22.79±4.7	<0.05
Utilization of social support	6.7±2.03	7.88±1.98	<0.05

### 3.5 Multivariate analysis of anxiety and depression in patients with KOA

A logistic regression analysis was conducted to examine the relationship between various factors and the incidence of anxiety and depression in patients with KOA. The analysis identified several significant independent variables influencing anxiety, including BMI, QOL, and utilization of social support. Specifically, lower BMI reduced QOL, and diminished utilization of social support were associated with an increased likelihood of anxiety among KOA patients (*P* < 0.05). In addition, the analysis revealed that age, BMI, QOL, function, and daily activities score, subjective support, and utilization of social support were significant factors affecting depression in this population. Younger age, lower BMI, poorer QOL, and lower levels of both subjective support and utilization of social support correlated with a higher probability of depression. Conversely, a higher score in function and daily activities was also linked to increased depression risk (*P* < 0.05). Detailed results can be found in **Tables 6** and **7**.

**Table 6:** Multivariate analysis of anxiety in patients with KOA.

	Regression coefficient	Standard error	Wald χ^2^	*P* value	OR*	95%CI
BMI	-0.579	0.253	5.227	<0.05	0.561	0.341-0.921
Education						
Junior high school or lower^a^						
High school or junior college	-0.104	0.855	0.015	0.903	0.901	0.169-4.186
University degree or higher	-0.285	0.865	0.109	0.742	0.752	0.138-0.496
Residence	-0.380	0.302	1.585	0.208	0.684	0.378-1.236
Professional status	-1.425	0.795	3.211	0.073	0.241	0.051-1.143
Quality of life	-6.677	1.942	11.820	<0.05	0.001	0.000-0.057
Stiff	-0.085	0.097	0.760	0.383	0.919	0.759-1.112
Pain	0.066	0.056	1.368	0.242	1.068	0.956-1.193
Function and daily activities	0.027	0.017	2.579	0.108	1.028	0.994-1.063
Utilization of social support	-0.174	0.067	6.749	<0.05	0.841	0.737-0.958

*Odds Ratio (OR) is a measure of the difference in the probability of an event occurring between two different groups, assessing the strength of the association between exposure to a factor and the occurrence of a disease; 95% confidence interval(CI)is a description of uncertainty about population parameters.

a represents a reference group.

**Table 7:** Multivariate analysis of depression in patients with KOA.

	Regression coefficient	Standard error	Wald χ^2^	*P* value	OR*	95%CI
Age	-0.466	0.184	6.382	<0.05	0.628	0.437-0.901
BMI	-0.532	0.253	4.430	<0.05	0.587	0.358-0.964
Education						
Junior high school or lower^a^						
High school or junior college	1.140	0.840	1.841	0.175	3.128	0.602-16.242
University degree or higher	0.642	0.835	0.591	0.442	1.901	0.370-9.775
Income	0.200	2.250	0.643	0.423	1.222	0.749-1.994
Residence	-0.162	0.321	0.254	0.614	0.851	0.453-1.596
Quality of life	-5.012	1.880	7.107	<0.05	0.007	0.000-0.265
Stiff	0.121	0.092	1.730	0.188	1.129	0.942-1.353
Function and daily activities	0.033	0.015	5.138	<0.05	1.034	1.004-1.064
Subjective support	-0.074	0.031	5.626	<0.05	0.929	0.874-0.987
Utilization of social support	-0.224	0.068	10.710	<0.05	0.800	0.699-0.914

*Odds Ratio (OR) is a measure of the difference in the probability of an event occurring between two different groups, assessing the strength of the association between exposure to a factor and the occurrence of a disease; 95% confidence interval (CI) is a description of uncertainty about population parameters.

a represents a reference group.

## 4 Discussion

In this study, the HADS was employed to assess anxiety and depression in patients with KOA. The objective was to understand these pathological emotions and explore their influencing factors to enhance clinical guidance and improve diagnostic and treatment strategies. The findings indicated that body BMI, QOL, and utilization of social support were negatively correlated with anxiety symptoms in KOA patients. Additionally, age, BMI, QOL, subjective support, and utilization of social support were negatively correlated with depressive symptoms, whereas functional and daily activity scores showed a positive correlation with depressive symptoms.

The global prevalence of osteoarthritis is approximately 30% (22). Among these patients, one in five experiences symptoms of anxiety and depression (23).KOA is associated with anxiety and depression, and several high-quality clinical trials have confirmed that the antidepressant Duloxetine can effectively relieve pain symptoms and improve knee function in patients with KOA. Therefore, some scholars propose that there may be a standard central mechanism between KOA and depression. Further research indicates that chronic pain leads to atrophy in brain regions responsible for regulating mood and managing emotions (24). This atrophy can disrupt emotional homeostasis, impairing the ability to respond to stressors. Consequently, KOA patients may feel isolated and experience a decline in self-efficacy, making them more susceptible to anxiety and depression (25, 26).

Our study revealed that among individuals over the age of 40, younger participants are more susceptible to depression. This increased likelihood of depression may be attributed to stressors related to family, work, and social environments. Additionally, patients with KOA who have a low BMI experience a diminished QOL due to poor nutritional status. This nutritional deficiency may contribute to a higher propensity for mood disorders in these patients compared to those with a higher BMI. These findings align with the research conducted by Hye (27). In addition, A meta-analysis revealed a significant correlation between depression and knee function in patients with KOA (28). Our findings support this correlation, indicating that patients with higher scores in activities of daily living are more susceptible to depression. Literature suggests that anxiety and depression can exacerbate pain symptoms in KOA patients, further limiting knee joint function. This cycle can lead to the emergence of additional psychological issues, ultimately diminishing patients’ QOL and intensifying depressive symptoms (29–32). Consequently, it is essential to address both clinical and psychological treatments simultaneously in the management of KOA patients to achieve optimal treatment outcomes. It should be noted that when exploring the relationship between pain symptoms and anxiety and depression, we did not observe meaningful results, which is inconsistent with previous research results (26, 33, 34). This discrepancy may be attributed to the clinical manifestations observed in KOA patients during this study, where symptoms such as pain, stiffness, and limited activity predominated. In contrast, fewer patients reported pain as the primary symptom. Consequently, the connection between pain and mood disorders warrants further investigation in future research.

An essential finding of this study is the significant correlation between anxiety, depression, and social support among patients with KOA. Social support refers to the emotional, material, and informational help and support that individuals receive from their social networks. Considerable attention has been given to its role in mediating somatic disability and depressive symptoms in the elderly (35). The impairment of daily activities in patients with KOA is recognized as a physical disability that significantly affects their QOL. This disability hinders their ability to perform basic self-care tasks and diminishes their capacity to engage with their social environment. Consequently, these limitations not only impact the physiological aspects of the patients but also have profound psychological effects, thereby elevating the risk of developing depressive symptoms (36). Adverse socioeconomic effects such as poverty, family breakdown, and emotional distress are also associated with depression (37). Research shows that social support can mitigate the adverse effects of physical disability on mental health. Conversely, patients with KOA are more likely to suffer from anxiety and depression when they lack adequate social support (38). Furthermore, our analysis revealed no multicollinearity between social support and QOL, indicating an absence of a significant correlation between these two variables. This finding suggests that the conclusions drawn in this study regarding the impact of social support and QOL on anxiety and depression are both stable and reliable. However, prior research has highlighted that different dimensions of social support may exert varying influences on health-related QOL. While this discrepancy may appear inconsistent with our findings, it is likely attributable to variations in research contexts, socioeconomic environments, measurement methodologies, and dataset characteristics (39). Thus, patients with KOA who lack sufficient social support often experience adverse psychological effects, including reduced self-esteem and limited social activities. These psychological challenges, in turn, heighten their need for social support and increase their vulnerability to depressive symptoms. So, when evaluating the reported outcomes of patients with KOA, it is essential to consider the level of social support they receive. This consideration is crucial, as the extent of social support can significantly influence the severity of self-reported symptoms and psychological issues (40). Additionally, studies have confirmed that patients residing in areas characterized by social deprivation, which is marked by low levels of social support, tend to report elevated depressive symptoms and diminished overall well-being (41–43). This highlights the interconnectedness of physical and mental health, suggesting that the social support level can significantly influence other domains. Consequently, establishing social support and positive psychological interventions is crucial for enhancing social support levels and improving the mental health of patients with KOA.

Our findings indicate significant mental health issues among patients with KOA, with high prevalence rates of anxiety and depression. A growing body of evidence underscores the detrimental effects of mental health problems on postoperative outcomes in KOA patients (44, 45), such as persistent pain and limited mobility diminishing the QOL for patients with KOA, warranting consideration by orthopedics doctors and experts when designing treatment plans or clinical pathways. Following diagnosis, a comprehensive approach that integrates pharmacological treatment, psychological therapy, counseling, and family support should be employed. This multifaceted strategy aims to enhance the quality of life for KOA patients and mitigate the incidence of anxiety and depression (46). Psychosocial interventions, including cognitive behavioral therapy and group acceptance and commitment therapy, play a crucial role in enhancing patient outcomes (47). These therapies can alleviate feelings of isolation and allow patients to learn from the experiences of others, thereby reducing anxiety and depression. Additionally, effective psychological interventions can diminish the pain experienced by patients with KOA. They also enhance self-efficacy, which in turn boosts both subjective and social support (48).

This study distinguishes itself from previous research, which primarily focused on general demographics and disease characteristics, by examining the relationship between QOL, social support levels, and the incidence of anxiety and depression among patients with KOA. It aims to determine whether enhancing QOL and social support can mitigate anxiety and depression, thereby offering a scientific basis for developing psychological intervention strategies. Furthermore, while existing research on the factors influencing anxiety and depression in KOA patients is more prevalent in the southern and eastern coastal regions of China, there is a lack of studies addressing these factors in the northern areas. This investigation and analysis of anxiety and depression in KOA patients from Taiyuan City and surrounding areas offer data to objectively evaluate these conditions and their influencing factors in northern China. The above contents are also the innovation of this study.

When conducting similar studies in the future, several considerations should be taken into account to enhance the research’s validity and applicability. First, increasing the sample size is crucial for improving the reliability of the study’s conclusions. Second, it is essential to conduct investigations across various periods, such as post-operation and post-discharge, to analyze the causes comprehensively and influencing factors of mental health issues in patients with KOA at different stages. This approach will enable medical professionals to develop targeted programs aimed at addressing the mental health challenges faced by KOA patients at each stage, thereby improving the overall quality of medical services. Third, incorporating imaging examinations for each participant can provide insights into the relationship between disease severity and the prevalence of anxiety and depression. Fourth, the conclusions drawn from observational studies could pave the way for future clinical trials, which would further investigate the efficacy of psychological interventions for KOA patients experiencing mood disorders. Finally, conducting multi-center research is recommended to facilitate comparisons of mental health levels among KOA patients in different regions. This comparison will offer readers a clearer understanding of the factors influencing anxiety and depression in this patient population.

In conclusion, factors such as age, BMI, QOL, subjective support, utilization of social support, and daily functioning significantly impact the mental health and QOL in patients with KOA. Therefore, to effectively manage patients with KOA, medical staff should optimize treatment plans by integrating clinical interventions aimed at alleviating symptoms and enhancing knee joint function. Additionally, establishing mechanisms for social support and positive psychological interventions is crucial to safeguarding elderly patients from depressive symptoms. By combining these approaches, the overall mental health and quality of life of KOA patients may be significantly improved.

Unfortunately, our study also has some limitations. Firstly, this study is a cross-sectional survey, which may have some recall bias and cannot provide direct causal evidence; thus, further experimental or longitudinal studies are necessary to facilitate an evaluation of causality. Secondly, the study was conducted at a single center. As a result, the findings are limited in scope and can only be generalized to the anxiety and depression experienced by KOA patients at this particular institution. Thirdly, the investigation was conducted prior to surgery, and no subsequent investigations or analyses were performed on KOA patients at various time points, such as postoperatively or at discharge. Fourthly, since some patients did not undergo an X-ray examination, the KL classification for KOA severity could not be determined for each patient. Consequently, this study did not analyze the relationship between disease severity and anxiety and depression. Finally, this study is observational, lacking any psychological interventions for KOA patients experiencing emotional disorders. Furthermore, no clinical trials were conducted to validate the accuracy of the conclusions or to assess the effectiveness of psychological interventions.

## Data Availability

The original contributions presented in the study are included in the article/supplementary material. Further inquiries can be directed to the corresponding author.

## References

[1] Sharma L. Osteoarthritis of the knee. New Engl J Med. (2021) 384:51–9. doi: 10.1056/NEJMcp1903768 33406330

[2] Kyriakidis T Asopa V Baums M Verdonk R Totlis T. Unicompartmental knee arthroplasty in patients under the age of 60 years provides excellent clinical outcomes and 10-year implant survival: a systematic review: a study performed by the Early Osteoarthritis group of ESSKA-European Knee Associates section. Knee Surgery Sports Traumatology Arthroscopy. (2023) 31:922–32. doi: 10.1007/s00167-022-07029-9 35763042

[3] Du X Liu ZY Tao XX Mei YL Zhou DQ Cheng K. Research progress on the pathogenesis of knee osteoarthritis. Orthopaedic Surg. (2023) 15:2213–24. doi: 10.1111/os.13809 PMC10475681 37435789

[4] Clark GP. Treatment options for symptomatic knee osteoarthritis in adults. Jaapa. (2023) 36:1–6. doi: 10.1097/01.JAA.0000979536.73946.98 37884044

[5] Moskowitz RW. The burden of osteoarthritis: clinical and quality-of-life issues. Am J managed Care. (2009) 15:S223–9. 19817508

[6] Nicholl BI Mackay D Cullen B Martin DJ Ul-Haq Z Mair FS. Chronic multisite pain in major depression and bipolar disorder: cross-sectional study of 149,611 participants in UK Biobank. BMC Psychiatry. (2014) 14:1–11. doi: 10.1186/s12888-014-0350-4 PMC4297369 25490859

[7] Stubbs B Eggermont L Mitchell AJ De Hert M Correll CU Soundy A. The prevalence of pain in bipolar disorder: a systematic review and large-scale meta-analysis. Acta Psychiatrica Scandinavica. (2015) 131:75–88. doi: 10.1111/acps.2014.131.issue-2 25098864

[8] Rathbun AM Harrold LR Reed GW. A description of patient-and rheumatologist-reported depression symptoms in an American rheumatoid arthritis registry population. Clin Exp Rheumatol. (2014) 32:523–32. 24984165

[9] Stubbs B Aluko Y Myint PK Smith TO. Prevalence of depressive symptoms and anxiety in osteoarthritis: a systematic review and meta-analysis. Age Ageing. (2016) 45:228–35. doi: 10.1093/ageing/afw001 26795974

[10] Farid AR Liimakka AP Parker EB Smith JT Melnic CM Chen AF. Association of pharmacologic treatment of depression/anxiety with initial patient-reported outcome measures in patients with hip and knee osteoarthritis. JAAOS-Journal Am Acad Orthopaedic Surgeons. (2024) 32:516–24. doi: 10.5435/JAAOS-D-23-00887 38595309

[11] Sorel JC Oosterhoff JHF Broekman BFP Jaarsma RL Doornberg JN Doornberg FF. Do symptoms of anxiety and/or depression and pain intensity before primary Total knee arthroplasty influence reason for revision? Results of an observational study from the Dutch arthroplasty register in 56,233 patients. Gen Hosp Psychiatry. (2022) 78:42–9. doi: 10.1016/j.genhosppsych.2022.07.001 35853417

[12] Dong Y Cai C Liu M Liu L Zhou F. Improvement and prognosis of anxiety and depression after total knee arthroplasty. Acta orthopaedica belgica. (2024) 90:211–6. doi: 10.52628/aob 39440495

[13] Zheng S Tu L Cicuttini F Zhu Z Han W Antony B. Depression in patients with knee osteoarthritis: risk factors and associations with joint symptoms. BMC musculoskeletal Disord. (2021) 22:1–10. doi: 10.1186/s12891-020-03875-1 PMC7791830 33413273

[14] Tsai YF. Gender differences in pain and depressive tendency among Chinese elders with knee osteoarthritis. Pain. (2007) 130:188–94. doi: 10.1016/j.pain.2007.03.014 17452080

[15] Kang HJ Bae KY Kim SW Shin HY Shin IS Yoon JS. Impact of anxiety and depression on physical health condition and disability in an elderly Korean population. Psychiatry Invest. (2017) 14:240. doi: 10.4306/pi.2017.14.3.240 PMC5440426 28539942

[16] Peeler J Ripat J. The effect of low-load exercise on joint pain, function, and activities of daily living in patients with knee osteoarthritis. Knee. (2018) 25:135–45. doi: 10.1016/j.knee.2017.12.003 29325839

[17] Lee AC Driban JB Price LL Harvey WF Rodday AM Wang C. Responsiveness and minimally important differences for 4 patient-reported outcomes measurement information system short forms: physical function, pain interference, depression, and anxiety in knee osteoarthritis. J Pain. (2017) 18:1096–110. doi: 10.1016/j.jpain.2017.05.001 PMC5581239 28501708

[18] Wilkie R Blagojevic-Bucknall M Jordan KP Lacey R McBeth J. Reasons why multimorbidity increases the risk of participation restriction in older adults with lower extremity osteoarthritis: a prospective cohort study in primary care. Arthritis Care Res. (2013) 65:910–9. doi: 10.1002/acr.21918 23225783

[19] Gandek B. Measurement properties of the Western Ontario and McMaster Universities Osteoarthritis Index: a systematic review. Arthritis Care Res. (2015) 67:216–29. doi: 10.1002/acr.22415 25048451

[20] Mykletun A Stordal E Dahl AA. Hospital Anxiety and Depression (HAD) scale: factor structure, item analyses and internal consistency in a large population. Br J Psychiatry. (2001) 179:540–4. doi: 10.1192/bjp.179.6.540 11731359

[21] Snaith RP. The hospital anxiety and depression scale. Health Qual Life outcomes. (2003) 1:1–4. doi: 10.1186/1477-7525-1-29 12914662 PMC183845

[22] Hiligsmann M Cooper C Arden N Boers M Branco JC Luisa Brandi M. Health economics in the field of osteoarthritis: an expert’s consensus paper from the European Society for Clinical and Economic Aspects of Osteoporosis and Osteoarthritis (ESCEO)[C//Seminars in arthritis and rheumatism. WB Saunders. (2013) 43:303–13. doi: 10.1016/j.semarthrit.2013.07.003 23992801

[23] Stubbs B Aluko Y Myint PK. Smith TO Prevalence of depressive symptoms and anxiety in osteoarthritis: a systematic review and meta-analysis. Age Ageing. (2016) 45:228–35. doi: 10.1093/ageing/afw001 26795974

[24] van der Esch M Knoop J van der Leeden M Roorda LD Lems WF Knol DL. Clinical phenotypes in patients with knee osteoarthritis: a study in the Amsterdam osteoarthritis cohort. Osteoarthritis cartilage. (2015) 23:544–9. doi: 10.1016/j.joca.2015.01.006 25596322

[25] de Rooij M van der Leeden M Heymans MW Holla JF Häkkinen A Lems WF. Course and predictors of pain and physical functioning in patients with hip osteoarthritis: Systematic review and meta-analysis. J Rehabil Med. (2016) 48:245–52. doi: 10.2340/16501977-2057 26871564

[26] van Dijk GM Dekker J Veenhof C van den Ende CH Carpa Study Group. Course of functional status and pain in osteoarthritis of the hip or knee: a systematic review of the literature. Arthritis Care Research: Off J Am Coll Rheumatol. (2006) 55:779–85. doi: 10.1002/art.22244 17013827

[27] Park HM Kim HS Lee YJ. Knee osteoarthritis and its association with mental health and health-related quality of life: a nationwide cross-sectional study. Geriatrics Gerontology Int. (2020) 20:379–83. doi: 10.1111/ggi.13879 32037727

[28] Fonseca-Rodrigues D Rodrigues A Martins T Pinto J Amorim D Almeida A. Correlation between pain severity and levels of anxiety and depression in osteoarthritis patients: a systematic review and meta-analysis. Rheumatology. (2022) 61:53–75. doi: 10.1093/rheumatology/keab512 34152386

[29] Rathbun AM Yau MS Shardell M Stuart EA Hochberg MC. Depressive symptoms and structural disease progression in knee osteoarthritis: data from the Osteoarthritis Initiative. Clin Rheumatol. (2017) 36:155–63. doi: 10.1007/s10067-016-3495-3 PMC5488696 27957619

[30] Burston JJ Valdes AM Woodhams SG Mapp PI Stocks J Watson DJG. The impact of anxiety on chronic musculoskeletal pain and the role of astrocyte activation. Pain. (2019) 160:658–69. doi: 10.1097/j.pain.0000000000001445 PMC6407811 30779717

[31] Iijima H Aoyama T Fukutani N Isho T Yamamoto Y Hiraoka M. Psychological health is associated with knee pain and physical function in patients with knee osteoarthritis: an exploratory cross-sectional study. BMC Psychol. (2018) 6:1–10. doi: 10.1186/s40359-018-0234-3 29716654 PMC5930799

[32] Kroenke K Wu J Bair MJ Krebs EE Damush TM Tu W. Reciprocal relationship between pain and depression: a 12-month longitudinal analysis in primary care. J Pain. (2011) 12:964–73. doi: 10.1016/j.jpain.2011.03.003 PMC3222454 21680251

[33] Liu M McCurry SM Belza B Dobra A Buchanan DT Vitiello MV. Effects of osteoarthritis pain and concurrent insomnia and depression on health care use in a primary care population of older adults. Arthritis Care Res. (2019) 71:748–57. doi: 10.1002/acr.2019.71.issue-6 PMC6358516 30067892

[34] de Koning EJ Timmermans EJ van Schoor NM Stubbs B van den Kommer TN Dennison EM. Within-person pain variability and mental health in older adults with osteoarthritis: an analysis across 6 European cohorts. J Pain. (2018) 19:690–8. doi: 10.1016/j.jpain.2018.02.006 PMC5975951 29496636

[35] Newsom JT Schulz R. Social support as a mediator in the relation between functional status and quality of life in older adults. Psychol Aging. (1996) 11:34. doi: 10.1037/0882-7974.11.1.34 8726368

[36] Lenze EJ Rogers JC Martire LM Mulsant BH Rollman BL Dew MA. The association of late-life depression and anxiety with physical disability: a review of the literature and prospectus for future research. Am J geriatric Psychiatry. (2001) 9:113–35. doi: 10.1097/00019442-200105000-00004 11316616

[37] Alexopoulos GS. Depression in the elderly. Lancet. (2005) 365:1961–70. doi: 10.1016/S0140-6736(05)66665-2 15936426

[38] Xie H Peng W Yang Y Zhang D Sun Y Wu M. Social support as a mediator of physical disability and depressive symptoms in Chinese elderly. Arch Psychiatr Nurs. (2018) 32:256–62. doi: 10.1016/j.apnu.2017.11.012 29579521

[39] Taylor MG Lynch SM. Trajectories of impairment, social support, and depressive symptoms in later life. Journals Gerontology Ser B: psychol Sci Soc Sci. (2004) 59:S238–46. doi: 10.1093/geronb/59.4.S238 15294928

[40] Wright MA Adelani M Dy C O’Keefe R Calfee RP. What is the impact of social deprivation on physical and mental health in orthopaedic patients? Clin Orthopaedics Related Research®. (2019) 477:1825–35. doi: 10.1097/CORR.0000000000000698 PMC7000003 31107333

[41] Kinderman P Tai S Pontin E Schwannauer M Jarman I Lisboa P. Causal and mediating factors for anxiety, depression and well-being. Br J Psychiatry. (2015) 206:456–60. doi: 10.1192/bjp.bp.114.147553 25858180

[42] Richardson R Westley T Gariépy G Austin N Nandi A. Neighborhood socioeconomic conditions and depression: a systematic review and meta-analysis. Soc Psychiatry Psychiatr Epidemiol. (2015) 50:1641–56. doi: 10.1007/s00127-015-1092-4 26164028

[43] Saito M Kondo K Kondo N Abe A Ojima T Suzuki K. Relative deprivation, poverty, and subjective health: JAGES cross-sectional study. PloS One. (2014) 9:e111169. doi: 10.1371/journal.pone.0111169 25350284 PMC4211701

[44] Khatib Y Madan A Naylor JM Harris IA. Do psychological factors predict poor outcome in patients undergoing TKA? A systematic review. Clin Orthopaedics Related Research®. (2015) 473:2630–8. doi: 10.1007/s11999-015-4234-9 PMC4488213 25791440

[45] Utrillas-Compaired A de la Torre-Escuredo BJ Tebar-Martínez AJ Asúnsolo-Del Barco Á. Does preoperative psychologic distress influence pain, function, and quality of life after TKA? Clin Orthopaedics Related Research®. (2014) 472:2457–65. doi: 10.1007/s11999-014-3570-5 PMC4079871 24671514

[46] Ferreira AH Godoy PB Oliveira NR Diniz RA Diniz RE Padovani Rda C. Investigation of depression, anxiety and quality of life in patients with knee osteoarthritis: a comparative study. Rev Bras reumatologia. (2015) 55:434–8. doi: 10.1016/j.rbr.2015.03.001 26198010

[47] Ferreira MG Mariano LI Rezende JV Caramelli P Kishita N. Effects of group Acceptance and Commitment Therapy (ACT) on anxiety and depressive symptoms in adults: A meta-analysis. J Affect Disord. (2022) 309:297–308. doi: 10.1016/j.jad.2022.04.134 35489560

[48] Zhang L Fu T Zhang Q Yin R Zhu L He Y. Effects of psychological interventions for patients with osteoarthritis: a systematic review and meta-analysis. Psychology Health Med. (2018) 23:1–17. doi: 10.1080/13548506.2017.1282160 28140653

